# Prolapsed Umbilical Meckel's Diverticulum Mimicking Umbilical Granuloma: A Rare Differential of a Common Problem

**DOI:** 10.7759/cureus.76351

**Published:** 2024-12-24

**Authors:** Muhammad M Saleem, Mishal Pervaiz, Uswah Shoaib, Ismail Mazhar, Gull Sher, Muhammad Ibrahim Tahir, Sehar Khauteja Khan, Muhammad Osama

**Affiliations:** 1 Pediatric Surgery, Combined Military Hospital Lahore, Lahore, PAK; 2 Anaesthesiology, Combined Military Hospital Lahore, Lahore, PAK; 3 Medicine, Lahore Medical and Dental College, Lahore, PAK; 4 General Surgery, Combined Military Hospital Lahore, Lahore, PAK

**Keywords:** gastrointestinal congenital anomaly, ileal prolapse, meckel´s diverticulum, omphalomesenteric duct, umbilical lesion

## Abstract

Umbilical lesions in children represent a wide spectrum of congenital or acquired anomalies. Congenital anomalies are mainly because of failed obliteration of the omphalomesenteric duct while acquired pathologies are either because of delayed umbilical cord separation causing umbilical granuloma or result from umbilical stump infection producing omphalitis with persistent discharge. Meckel's diverticulum is considered the most common gastrointestinal congenital anomaly resulting from obliteration failure of the omphalomesenteric duct while umbilical granuloma is a common acquired umbilical lesion seen in daily practice. Prolapsed Meckel's diverticulum mimicking an umbilical granuloma is rare; we report a case where it was misdiagnosed and mistreated as an umbilical granuloma based on its appearance.

## Introduction

The omphalomesenteric duct (OMD) provides communication between the embryonic yolk sac and midgut, with natural obliteration occurring around the fifth to seventh week of gestation. However, in around 2%-4% of the population, this obliteration process is incomplete, leading to a spectrum of lesions in the gastrointestinal system or around the umbilicus [1]. Meckel’s diverticulum is the most common gastrointestinal anomaly resulting from partial obliteration of the omphalomesenteric duct [2]. Meckel's diverticulum presents a wide spectrum of manifestations, ranging from asymptomatic cases (most common) to complications such as intestinal obstruction (volvulus or intussusception), gastrointestinal bleeding due to ectopic gastric mucosa, pneumoperitoneum from perforation, and, rarely, tumor formation [3]. Additionally, the persistence of the omphalomesenteric duct, also known as a patent vitelline intestinal duct (VID), may present with complications such as mucoid or fecal umbilical discharge, requiring careful clinical evaluation for differential diagnoses. An umbilical granuloma, often mischaracterized as a granuloma, is a congenital anomaly resulting from ectopic mucosa of a minor persistent VID. It is the most common acquired umbilical lesion in neonates, typically presenting as a polypoid mass with chronic mucoid discharge noticed by parents after cord separation [4]. Prolapse of Meckel's diverticulum, mimicking an umbilical granuloma in an infant, is a rare occurrence that has been sparsely reported in the literature. We present a case where prolapsed Meckel's diverticulum mucosa through the umbilicus was initially misdiagnosed as an umbilical granuloma, with no response to typical granuloma treatment.

## Case presentation

**Figure 1:**
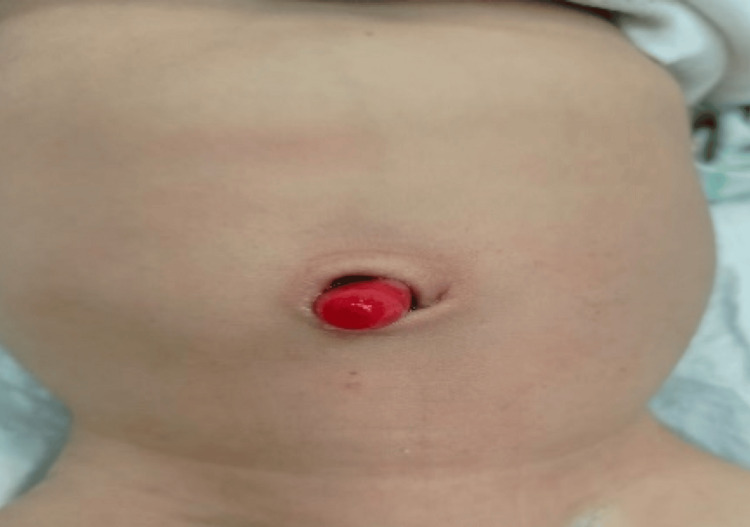
Appearance of umbilical mass on presentation.

A two-month-old boy with a birth weight of 2.7 kg, born by lower segment caesarian section (LSCS) at full-term gestation, was referred to the pediatric surgery outpatient unit with a history of mucoid discharge and polypoid growth protruding through the umbilicus noted by parents on separation of the umbilical cord at 12th day of neonatal life. The mother had regular antenatal check-ups and anomaly scans were unremarkable with no significant antenatal history. The child cried immediately after birth, tolerated initial feeds, and passed meconium through the anus. On separation of the umbilical stump, the mother noticed a red mass with a white discharge coming out from the umbilicus. There was no history of feculent discharge through the umbilicus. The size of the mass was initially small and gradually increased in size. Parents reported to a pediatrician with the same complaints and were advised topical silver nitrate gel. Ultrasound of the abdomen done before the start of treatment did not reveal any underlying communication. The mother continued the prescribed treatment for one month but there was no improvement in symptoms like mucoid discharge. Examination revealed a thriving, healthy child with normal weight for his age. Abdominal wall examination did not reveal any pathology except a 2x2 cm pinkish growth in the central part of the umbilicus (Figure 1).

**Figure 2:**
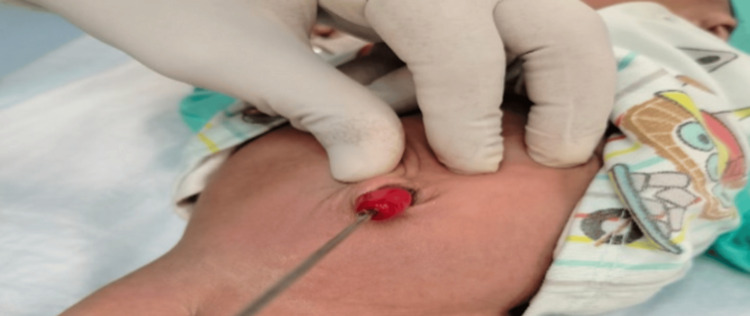
Gentle probing revealed free entry into peritoneal cavity.

On reviewing the baby, a small central aperture was noted in the mass. Gentle probing of the aperture revealed a free entrance inside the Meckel's lumen (Figure 2). The rest of the general physical and systemic examination was unremarkable. The abdomen was soft and nondistended, with audible bowel sounds. The anal opening was normally placed and patent. The mother reported no concerns regarding urinary or bowel issues.

**Figure 3:**
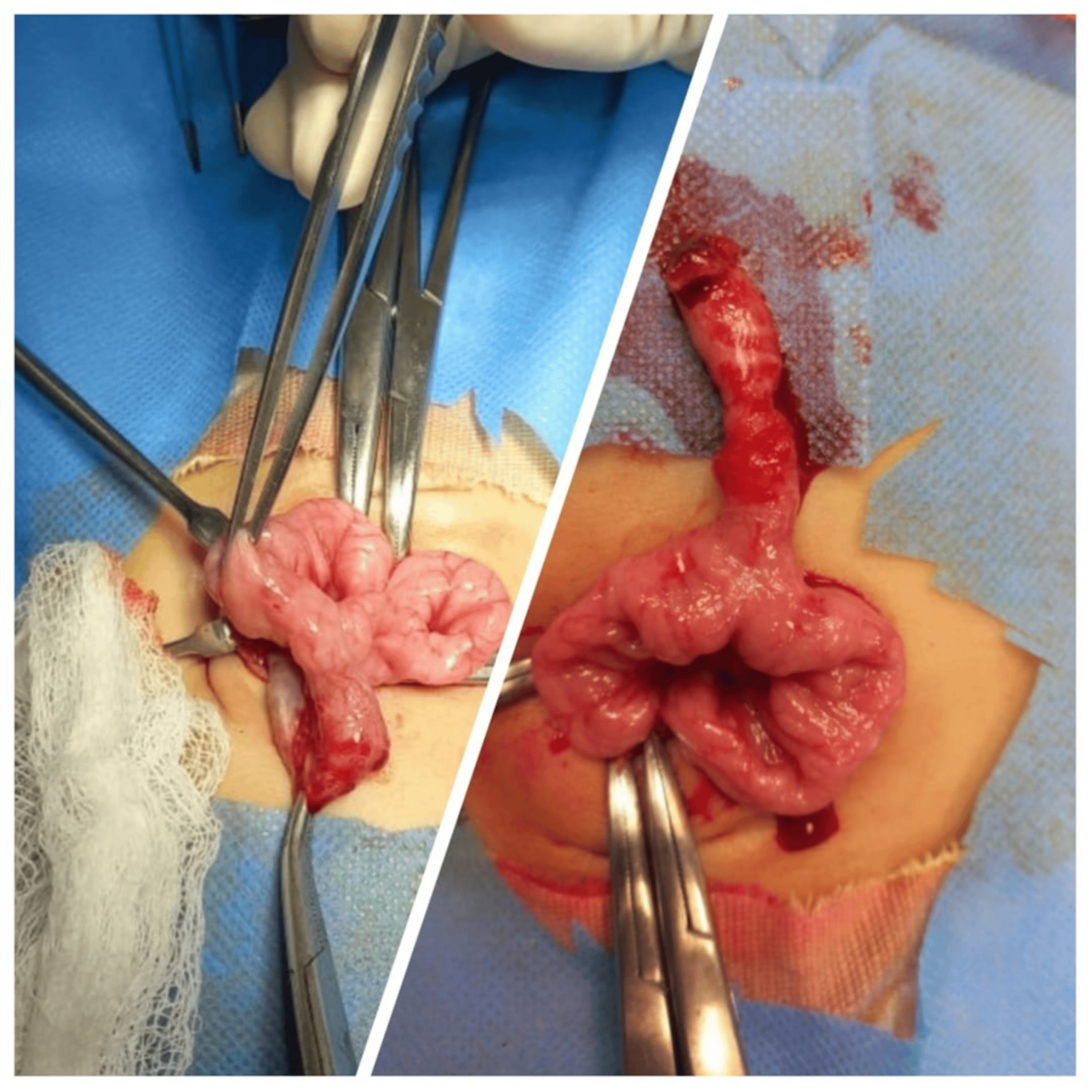
Intraoperative pictures showing prolapsed Meckel's diverticulum through umbilicus and following complete retrieval of specimen from umbilical stump after prolapsed mucosal inversion.

A provisional diagnosis of the patent omphalomesenteric duct was made, and laparotomy was performed which was done through a transverse incision encircling the umbilicus. Meckel's diverticulum of about 4cm in length was found intraoperatively with patent proximal and distal ileal limbs around 30cm from the ileocecal junction (Figure 3). Resection with primary end-to-end anastomosis was done using absorbable sutures to restore gut continuity. Umbilicoplasty was performed. The patient showed an uneventful recovery and was discharged on the sixth postoperative day. Serial follow-ups were scheduled at two weeks, one month, and two months which showed a thriving infant with well-healed scar and cosmetically satisfactory appearing umbilical stump.

**Figure 4:**
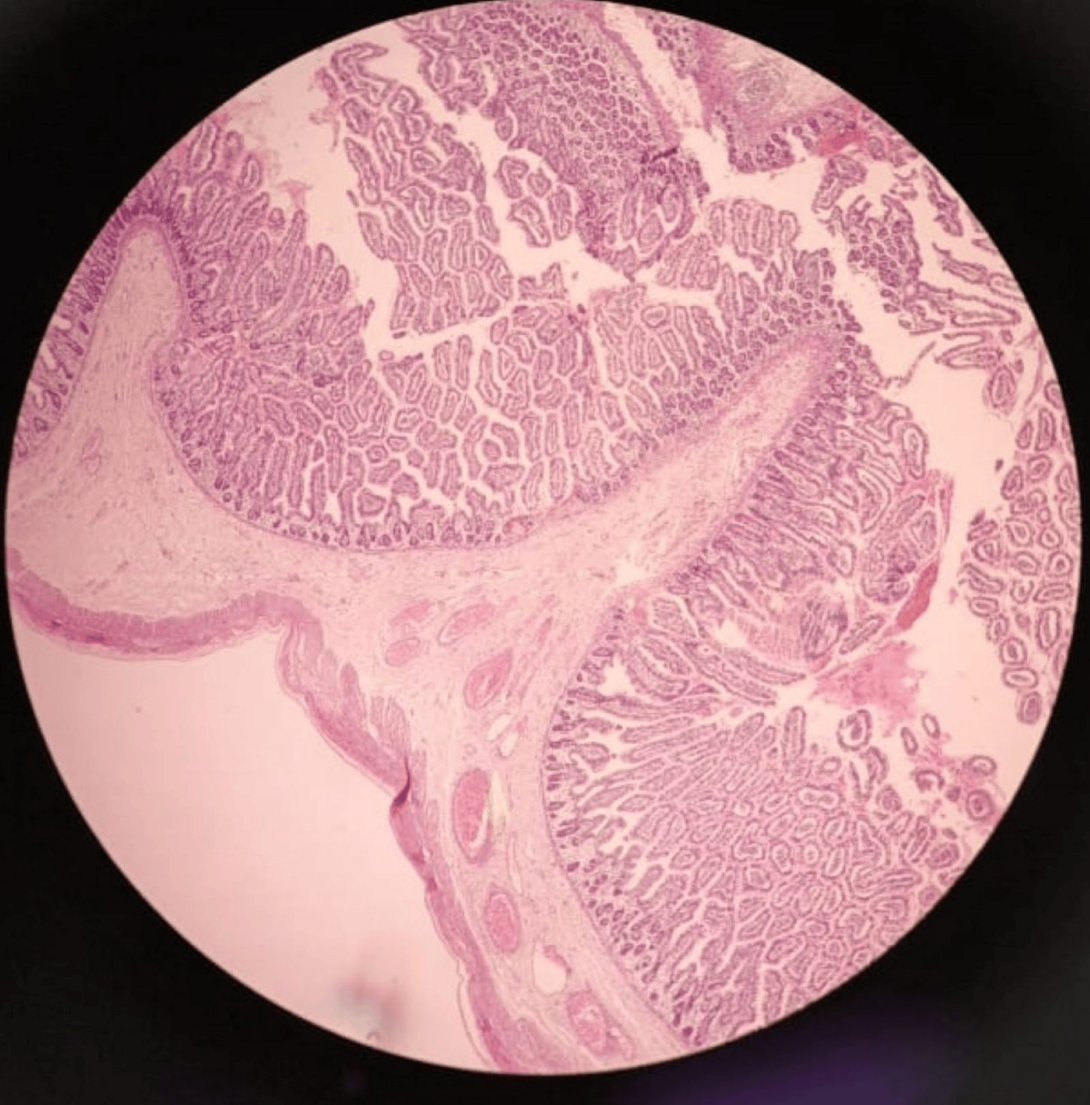
The sections from diverticulum reveal all layers of gut comprising of mucosa, submucosa, muscularis propria and serosa.

The histopathology report was consistent with Meckel’s diverticulum containing all layers of the intestinal wall (Figure 4).

## Discussion

The most frequent cause of umbilical discharge in pediatric patients is an umbilical granuloma, typically treated with silver application. Other important differentials of umbilical discharge are OMD remnants or a patent urachus. Ultrasound is usually the first investigation of choice that helps identify any communication between umbilical lesions and the intestine. In our case, the ultrasound findings were unremarkable, making it a less sensitive tool to pick up such communication. Our case involved a completely patent omphalomesenteric duct with a prolapsed Meckel's diverticulum, making it an exceptionally rare presentation. A literature review revealed no previous reports of Meckel's diverticulum prolapse, making our case the first to be documented.

Research indicates that congenital defects of the OMD are present in approximately 15% of the population, with a higher prevalence among males. These anomalies exhibit varied presentations, including Meckel’s diverticulum, VID, fistulas, sinuses, and cysts. Meckel’s diverticulum occurs in about 2% of the population, while complete patency of the omphalomesenteric duct is extremely rare, with an estimated incidence of 0.0053%, while prolapse through this patent duct is even rarer [5]. As our case is unique and previously unreported, we compared it to a patent VID with ileal prolapse. This type of prolapse is typically triggered by a sudden rise in intra-abdominal pressure caused by straining or crying in neonates [6]. Complete patency of VID remnants was first reported by Gvalani AK et al. in 1985 [7]. To date, very limited cases of ileal prolapse through a patent VID have been reported in the literature [8]. This condition constitutes a neonatal emergency because of the risk of complications, including intestinal obstruction, intussusception, strangulation, and gangrene of the prolapsed intestinal segment [6]. The prognosis depends on factors like the timing of presentation, prompt diagnosis, the presence of associated anomalies, and the size of the defect. In children presenting with mucoid or fecal umbilical discharge, a patent VID should be considered in the differential diagnosis, and a thorough clinical examination is essential to rule it out. In our case, there was no fecal discharge on presentation as the ileal luminal patency was preserved with only mucosal Meckel’s diverticulum prolapse through a patent duct.

The preferred treatment for patent VID with ileal prolapse is complete surgical excision of the patent VID and establishing the gut continuity with resection and primary anastomosis, as was in our case. A smiley periumbilical incision is the most commonly utilized approach for managing intestinal prolapse associated with a patent VID [1]. In cases of delayed presentation with complications, more aggressive surgical techniques such as formal laparotomy or ileostomy may be necessary [9].

## Conclusions

In conclusion, Meckel’s diverticulum with a patent VID presenting as an umbilical lesion is an exceptionally rare occurrence. This case underscores the limitations of ultrasound in detecting such anomalies and highlights the importance of a comprehensive preoperative evaluation to rule out VID patency in umbilical abnormalities. Surgeons must maintain a high index of suspicion for ileal prolapse and patent VID, as these conditions require distinct management approaches compared to more common diagnoses such as umbilical granulomas.
